# A Case Report Highlighting the Significance of COVID-19 Unveiling Megaloblastic Anemia and Worsening Dementia in the Elderly

**DOI:** 10.7759/cureus.62836

**Published:** 2024-06-21

**Authors:** Pooja Reddy, Pradeep Kumar Devarakonda, Gregory Gotlieb, Pedro Moreno

**Affiliations:** 1 College of Medicine, Georgetown University, Washington, D.C., USA; 2 Cardiology, Icahn School of Medicine at Mount Sinai, New York City, USA; 3 College of Medicine, University of Massachusetts Amherst, Amherst, USA; 4 Cardiology, Mount Sinai Hospital, New York City, USA

**Keywords:** covid-19, covid-19 impact of lockdown, elderly patients, food fortification, alzheimer’s dementia, dementia, megaloblastic anemia

## Abstract

The COVID-19 pandemic has resulted in substantial lifestyle changes with significant implications for nutritional health. Factors such as movement restrictions and disruptions in food supply chains led to the restricted availability of primary sources of essential micronutrients. To highlight this, we present the case of an elderly woman with an underlying subclinical cobalamin deficiency who developed symptomatic megaloblastic anemia, requiring hospital admission under lockdown conditions. This exemplifies how changes in diet during the COVID-19 lockdown have hastened the onset of B12 deficiency symptoms. Adverse outcomes can be avoided by identifying people at high risk of poor nutritional status and implementing policy initiatives that enhance their nutritional condition. This case report showed how important the B12 shortage was during the COVID-19 lockdown, especially for older people. They are more likely to be malnourished during COVID-19 for several reasons.

## Introduction

Vitamin B12 (cobalamin) plays a vital role in DNA synthesis, cellular metabolism, and myelin sheath maintenance, and its deficiency can lead to significant clinical implications. Previous reports suggested that the prevalence of vitamin B12 deficiency with classic hematological and neurological manifestations is low. However, B12 deficiency appears to be common among the elderly, affecting up to 26% of the elderly population [1]. Patients with severe vitamin B12 deficiency can present with variable manifestations, including megaloblastic anemia, weight loss, anorexia, paresthesia, and, in the most severe form, irreversible cognitive impairment and memory loss [2,3]. In older adults, B12 deficiency can mimic symptoms of age-related cognitive decline and may go undetected [4]. Particularly, enforced lockdown measures during COVID-19 have disrupted food systems and dietary practices, potentially inducing micronutrient deficiencies [5]. Factors such as movement restrictions and disruptions in food supply chains led to restricted availability of primary sources of essential micronutrients [6]. In addition, the fear of infection, compulsory stay-at-home orders, stress, and anxiety might have led to significant changes in dietary behaviors towards less consumption of nutrient-rich foods, subsequently increasing the risk of micronutrient deficiencies [5]. Previous reports showed a notably high prevalence of micronutrient deficiencies (79%) in elderly patients hospitalized with COVID-19 [7].

This case report describes how COVID-19 lockdown measures in 2020, from March to July, triggered the development of symptomatic megaloblastic anemia and worsening dementia in an elderly woman.

## Case presentation

**Figure 1:**
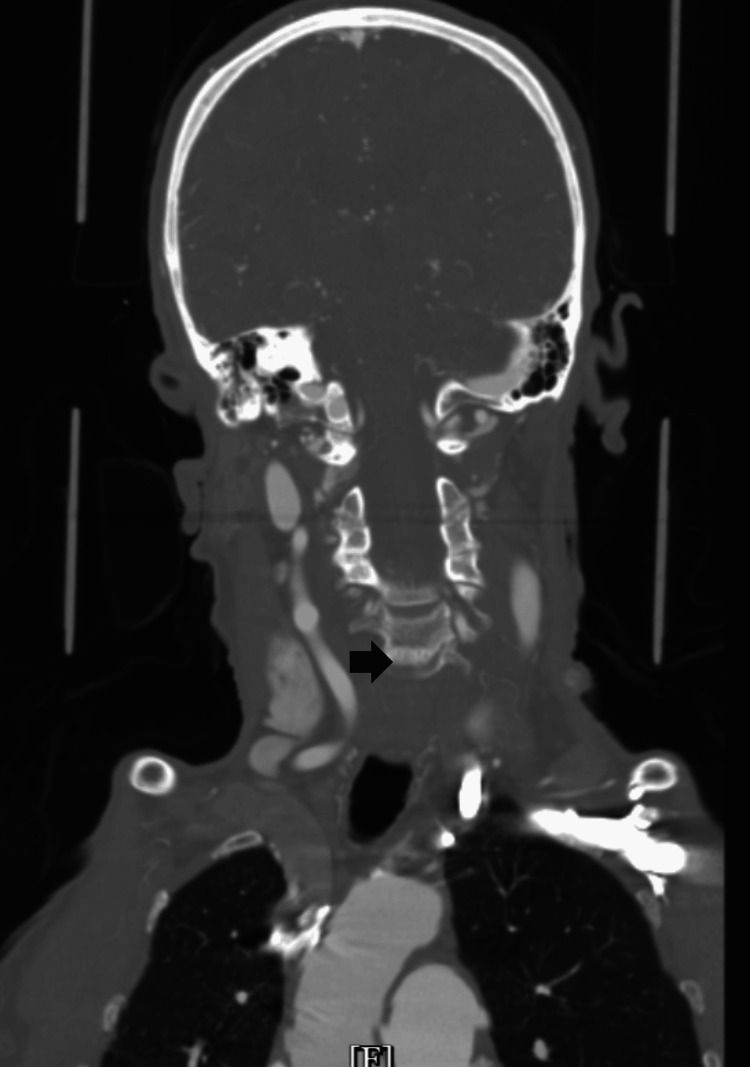
CTA showing occluded common and internal carotid on the left side (indicated by the black arrow, with the right side showing a patent carotid vessel). CTA: computed tomography angiography

An 88-year-old female patient with a known history of stroke, occlusion of the left carotid artery, and mild dementia presented to the Emergency Department (ED) complaining of decreased appetite, increased fatigue, and ambulatory difficulties. Her son reported notable changes in her behavior, including diminished food intake, excessive sleep, and imbalanced walking due to an unsteady sensation in her feet and worsening dementia. Upon evaluation, the patient demonstrated altered cognition and an inability to give a detailed medical history. We initially suspected a cognitive decline related to her history of previous strokes, and the patient was admitted to the cardiac telemetry unit. A carotid computed tomography angiography (CTA) confirmed the occlusion of the left common and internal carotid arteries, consistent with her previous history (Figure 1). A brain magnetic resonance imaging (MRI) scan did not show any acute infracts.

**Figure 2:**
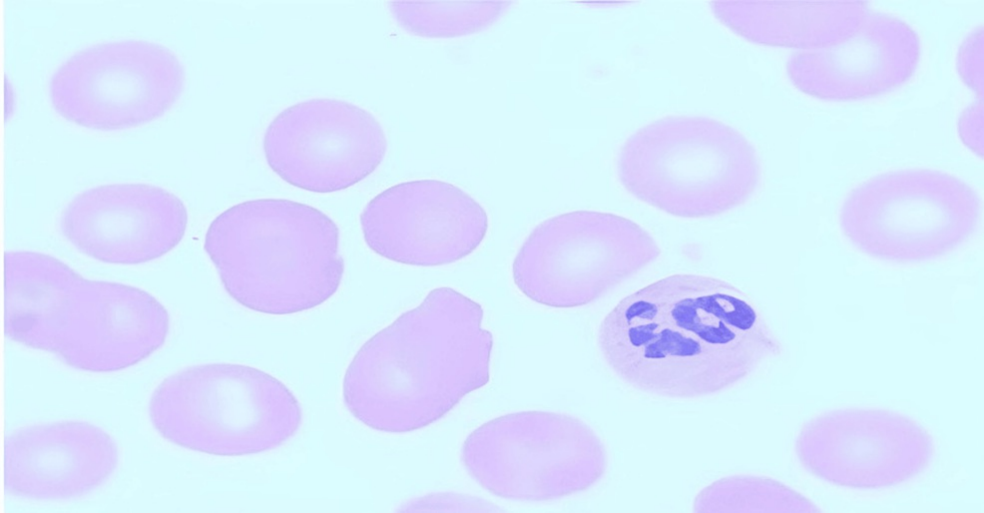
Peripheral smear with macrocytes and hyper-segmented neutrophil (magnification - 100X).

Subsequent laboratory investigations revealed the presence of megaloblastic anemia, indicated by a drop in hemoglobin levels to 7 g/dL, compared to 11.4 g/dL six months prior, a reticulocyte count of 0.8%, and a mean corpuscular volume (MCV) of 125 fL (Figure 2). Further assessment of her B12 and folate levels revealed a significant B12 deficiency, with a level of 109 pg/mL (normal range: 200 pg/mL to 900 pg/mL) (Table 1).

**Table 1:** Patient laboratory values upon presentation. g/dL: grams per deciliter; fL: femtoliter; pg/mL: picograms per milliliter; ng/mL: nanograms per milliliter; mg/dL: milligrams per decilitre

Laboratory test	Patient values	Reference normal range
Hemoglobin levels	7 g/dL	11.5-14.1 g/dL
Reticulocyte count	0.8%	1-2%
Mean corpuscular volume (MCV)	125 fL	80-94 fL
B12 level	109 pg/mL	200 pg/mL to 900 pg/mL
Serum folate	5.9 ng/mL	>5.4 ng/mL
Total bilirubin	0.3 mg/dL	0.2-1.2 mg/dL

A thorough food history revealed a limited diet devoid of dairy, eggs, and meat and mostly composed of vegetables, likely as a result of COVID-19 lockdown limitations. A B12 shortage was probably caused by this restricted diet in addition to a progressive loss of appetite.

The patient was managed with intramuscular injections of vitamin B12 and subsequent oral supplementation. This treatment notably improved her serum B12 levels to 400 pg/mL, memory improved and ameliorated her symptoms. Upon discharge, the patient received comprehensive dietary education and appropriate nutritional supplements.

## Discussion

Vitamin B12, also known as cobalamin, is an essential water-soluble vitamin with significant physiological roles in the human body. It is primarily derived from animal-based food sources. Rich sources include liver, clams, sardines, beef, fortified cereals, and dairy products [8]. The vitamin is synthesized exclusively by certain bacteria and is found in animal products due to the bacteria's symbiotic relationship within the animal's gastrointestinal tract. The absorption process of vitamin B12 is complex and involves multiple steps. When ingested, it binds to a protein called haptocorrin (also known as R protein) present in the saliva and gastric juice. In the acidic environment of the stomach, the R protein is removed, and the free cobalamin binds to the intrinsic factor (IF). The distal ileum absorbs the IF-cobalamin complex into the bloodstream through specific receptors. Once absorbed, B12 is transported in the bloodstream bound to transcobalamin, forming holotranscobalamin, which is then stored in the mitochondria [9]. Vitamin B12 facilitates the conversion of homocysteine to methionine, an essential amino acid. Methionine is then further converted into S-adenosylmethionine (SAM), which serves as a methyl donor for numerous methylation reactions, including the methylation of DNA. Vitamin B12 is also a crucial component of the enzyme methyl malonyl-CoA mutase, which converts methyl malonyl-CoA to succinyl-CoA, an important step in the breakdown of certain amino acids and lipids. Furthermore, B12 is essential for maintaining the myelin sheath surrounding neurons [10,11]. In return, vitamin B12 deficiency can lead to various clinical, particularly neurological, complications [12].

Vitamin B12 deficiency can arise from several factors that broadly fall into two categories: decreased intake and impaired absorption. Impaired absorption of vitamin B12 is the most common cause of deficiency, resulting from pernicious anemia, atrophic gastritis, gastrointestinal resection, celiac disease, or medications [13]. In addition, vitamin B12 deficiency can also develop from a poor intake of animal-based food sources or malnutrition [14]. In particular, elderly people often suffer from malnutrition and inadequate food intake, increasing their risk of vitamin B12 deficiency. In addition, atrophic gastritis and protein-bound malabsorption are common in the elderly, which can lead to vitamin B12 deficiency [10,15]. In our case, the patient had a history of stroke and dementia, potentially precipitating an increased risk of vitamin B12 deficiency.

Although vitamin B12 deficiency due to limited nutritional intake is rare, we believe that the COVID-19 lockdown measures further exacerbate the underlying subclinical vitamin B12 insufficiency in our case and induced neurological manifestations. Several factors can explain the role of the COVID-19 lockdown measures in developing clinical manifestations of the present case. The COVID-19 pandemic and associated lockdown measures have had a significant, disproportionate impact on the elderly population, particularly regarding nutritional inadequacy. The physiological changes associated with aging, such as impaired mastication and swallowing, alterations in taste, cognitive decline, and decreased mobility, inherently predispose this population to nutritional challenges [16]. The restrictions imposed during the pandemic might have amplified these vulnerabilities. Socioeconomic factors during lockdowns further hinder the elderly's ability to maintain adequate nutrition. Food insecurity has surged due to reduced access, limited availability, escalated food prices, insufficient social support for food procurement, and the relative poverty frequently encountered by the elderly [17]. Additionally, the fear of COVID-19 infection, isolation from family and friends, loss of independence, and loneliness have exacerbated depression and anxiety, consequently impacting dietary behaviors and nutritional intake [18].

Another potential explanation for our findings is the impact of COVID-19 on dietary habits. Although the COVID-19 lockdown had beneficial effects on eating practices such as home cooking, previous reports also suggested that the lockdown was associated with negative eating habits. Specifically, the consumption of snacks, potato chips, soups, and alcohol increased, and the intake of fresh foods, meat, and dairy declined. An elevated preference for comfort foods and alcohol was also noted. These unfavorable alterations in dietary practices were largely attributed to limited food availability and increased food prices [18]. Thus, future lockdowns should be associated with short- and long-term policies to maintain good lifestyle habits and minimize long-term health impacts.

Extensive research has demonstrated the lasting impact of SARS-CoV-2 infections on neurological functions, including memory and cognition. A study by Xie et al. highlighted mental health issues in COVID survivors, while another analysis revealed that one million Americans suffered from significant memory and concentration problems during the pandemic. Furthermore, human brain research has unveiled accelerated aging, abnormal structural changes, and prolonged neuroinflammatory reactions as a result of COVID-19. These findings suggest that impaired cognition following SARS-CoV-2 infection may be linked to a dysfunctional hypothalamic-pituitary response and reduced serotonin-induced vagal signaling [19,20].

The present case report carries several healthcare and social policy implications. Prior to the pandemic, strategies such as enhancing public transportation, increasing the accessibility of high-quality, affordable food items in local supermarkets, and facilitating participation in food assistance programs were recommended for addressing food insecurity. These strategies remain pivotal in confronting pandemics such as COVID-19. As described, the COVID-19 pandemic has disproportionately impacted elderly individuals. This underlines the need to address the socioeconomic determinants of food availability and dietary quality. Social distancing measures have inadvertently compounded pandemic-related food poverty by restricting access to food security services for elderly individuals. Efforts to alleviate these economic and physical barriers can take several forms, including supplemental financial assistance for food and bills, waiving delivery fees, and providing information and assistance in applying for food assistance programs. Community meal services have benefited the elderly during the COVID-19 lockdown [21]. Lastly, strategies should be developed, potentially through biofortification, vitamin supplementation, or government policies, to enhance access to meat and fish among the elderly. Adverse outcomes can be prevented by identifying individuals at high risk of malnutrition and implementing policy initiatives to improve their nutritional status [5].

## Conclusions

This case study brought to light the significance of B12 deficiency during the COVID-19 pandemic and its disproportionate effect on the elderly population, specifically inadequate nutrition. As mentioned before, there are numerous reasons why the elderly population is more susceptible to malnutrition during COVID-19. Strategies for this high-risk population need to be created and put into action to prevent these outcomes. This case report also emphasizes the significance of addressing the socioeconomic factors that influence food accessibility and nutrition quality.
